# Decoding microRNA drivers in atherosclerosis

**DOI:** 10.1042/BSR20212355

**Published:** 2022-07-15

**Authors:** Tanwi Vartak, Soundharya Kumaresan, Eoin Brennan

**Affiliations:** Diabetes Complications Research Centre, Conway Institute and School of Medicine, University College Dublin, Dublin, Ireland

**Keywords:** atherosclerosis, endothelial cells, microRNA, non-coding RNA, vascular smooth muscle

## Abstract

An estimated 97% of the human genome consists of non-protein-coding sequences. As our understanding of genome regulation improves, this has led to the characterization of a diverse array of non-coding RNAs (ncRNA). Among these, micro-RNAs (miRNAs) belong to the short ncRNA class (22–25 nucleotides in length), with approximately 2500 miRNA genes encoded within the human genome. From a therapeutic perspective, there is interest in exploiting miRNA as biomarkers of disease progression and response to treatments, as well as miRNA mimics/repressors as novel medicines. miRNA have emerged as an important class of RNA master regulators with important roles identified in the pathogenesis of atherosclerotic cardiovascular disease. Atherosclerosis is characterized by a chronic inflammatory build-up, driven largely by low-density lipoprotein cholesterol accumulation within the artery wall and vascular injury, including endothelial dysfunction, leukocyte recruitment and vascular remodelling. Conventional therapy focuses on lifestyle interventions, blood pressure-lowering medications, high-intensity statin therapy and antiplatelet agents. However, a significant proportion of patients remain at increased risk of cardiovascular disease. This continued cardiovascular risk is referred to as residual risk. Hence, a new drug class targeting atherosclerosis could synergise with existing therapies to optimise outcomes. Here, we review our current understanding of the role of ncRNA, with a focus on miRNA, in the development and progression of atherosclerosis, highlighting novel biological mechanisms and therapeutic avenues.

## Introduction

**Figure 1:**
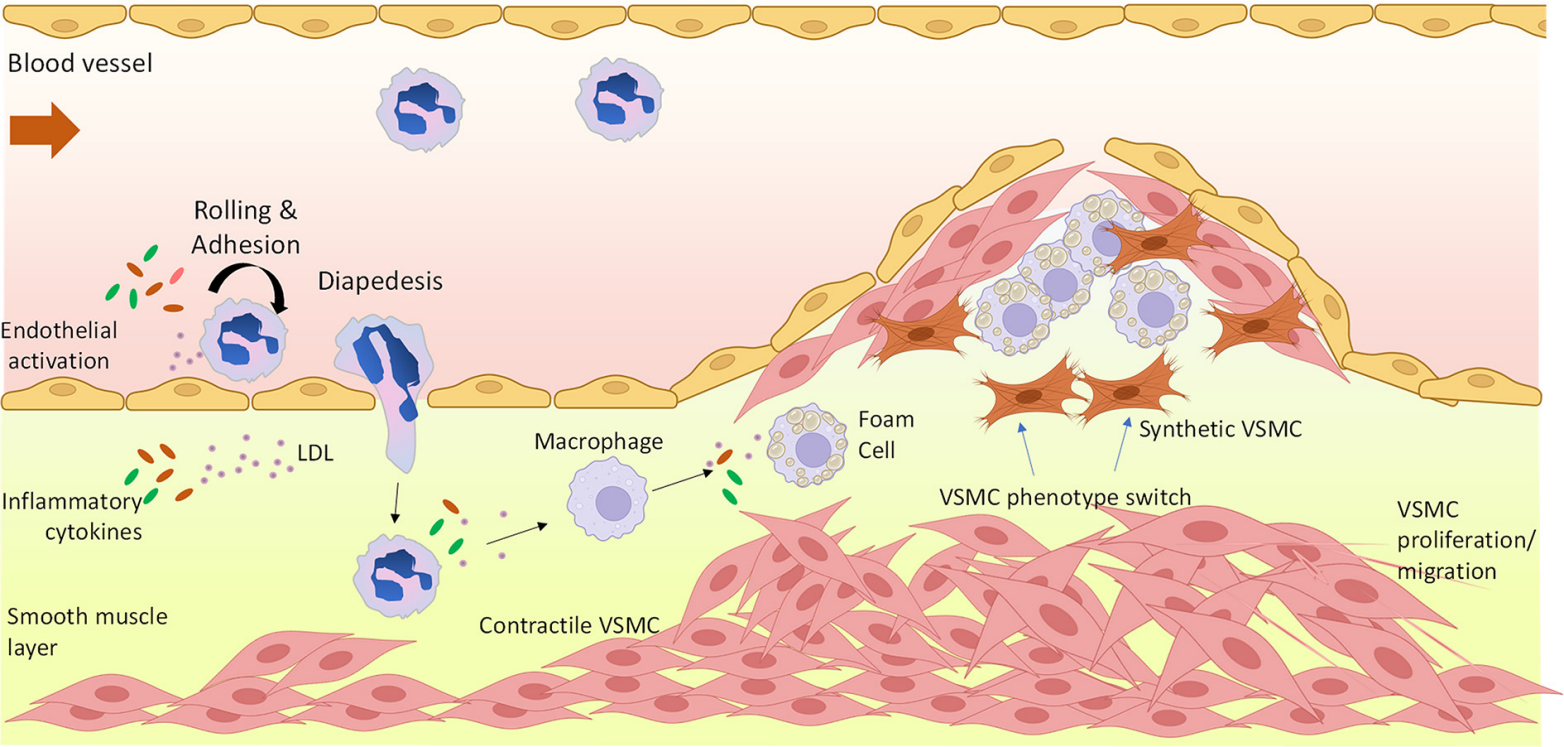
Pathogenesis of atherosclerosis Atherosclerotic lesions develop at the site of vascular endothelium damage, leading to up-regulation of vascular adhesion molecules (e.g. VCAM1, ICAM) and expression of monocyte chemoattractants [monocyte chemoattractant protein-1 (e.g. MCP-1/CCL2)]. Together, these facilitate leukocyte migration and adhesion. Smooth muscle cells (vSMCs) proliferate and migrate to the lesion, undergoing a defifferentiation from contractile to synthetic SMCs. Recent studies investigating SMC plasticity during atherosclerosis suggest that SMC phenotypic switching can contribute multiple cell-types within the lesion, including macrophage-like cells, foam-cell like cells, myofibroblast-like cells and mesenchymal stem cell like cells. In response to proatherogenic stimuli within the vessel wall, macrophages transform into lipid-laden foam cells which significantly contribute to lesion progression. T-cell activation and platelet adherence also occurs. Plaque rupture occurs in advanced lesions as a result of fibrous cap thinning. Created with BioRender.com.

Cardiovascular disease (CVD) remains a leading killer around the globe ending the lives of approximately 17 million people a year [1,2]. Dyslipidemia is defined as an imbalance of circulating lipid levels promoting the development of atherosclerosis. These lipid abnormalities include increased low-density lipoprotein cholesterol (LDL-C), decreased high-density lipoprotein cholesterol (HDL-C), and increased serum triglycerides. LDL-C accumulation within the artery wall is a central driver of atherosclerotic CVD (ASCVD). Immune cell infiltration, hyperlipidemia and inflammatory cytokines promote oxidative stress and endothelial cell (EC) dysfunction, leading to the trapping and oxidation of lipoproteins within the intimal layer of the vascular wall [2,3]. If untreated, a fibromuscular plaque can develop, which may destabilize, rupture and hemorrhage, resulting in major adverse cardiovascular events including myocardial infarction and stroke (Figure 1). At a cellular level, hemodynamic stresses, high-fat diet and elevated inflammatory cytokines promote vascular endothelium damage and dysfunction, resulting in elevated lipid accumulation in the vessel wall and atherosclerotic plaque development. The endothelium is, thus, an important target for therapies. Arterial macrophages infiltrate the vascular intimal layer, accumulating excessive lipoprotein, thus forming foam cells and contributing to plaque development. Foam cells are major contributors of necrotic core formation in atherosclerotic plaques, and therapies targeting foam cells are of significant interest. Underlying the vessel wall, the vascular smooth muscle cell (vSMC) layer plays a significant role in plaque development, progression, and stability through phenotypic switching, a pathological process in which vSMCs dedifferentiate, migrate, and transdifferentiate into other cell types. Yet how vSMCs contribute to the pathophysiology of atherosclerosis remains elusive, and there is great interest in understanding the plasticity of vSMCs as they dedifferentiate from a quiescent, contractile phenotype to a more synthetic, proinflammatory, pro-proliferative and promigratory phenotype [2,4]. Thus, directly targeting vSMCs during atherosclerosis provides therapeutic promise.

Conventional therapy focuses on lifestyle interventions, blood pressure-lowering medications, high-intensity statin therapy and antiplatelet agents (Figure 3). Statins remain the most common therapeutic agent for lipid lowering through the inhibition of 3-hydroxy-3-methylglutaryl coenzyme A reductase [5–8]. The pleiotropic effects of statins are well described in the literature, with effects believed to extend beyond lipid lowering, including direct anti-inflammatory activity, improved endothelial function and stabilization of high-risk plaques [5,9–12]. Despite these beneficial effects, a significant proportion of statin-treated patients remain at substantial risk of CVD [6,13–16]. This continued residual cardiovascular risk after optimal LDL-C control persists in some individuals for many reasons, including non-tolerance of statins due to adverse effects, poor adherence to medications, and requirements for patient-specific LDL-C targets rather than broader clinical subgroup LDL-C targets. In this regard, further reductions in LDL-C are achievable via the use of proprotein convertase subtilisin/kexin type 9 (PCSK9) inhibitors, which act to increase the recycling of LDL receptors in the liver, thereby lowering LDL levels [17,18]. Non-LDL-C risk factors also contribute to ASCVD, including levels of VLDL-C (very low-density lipoprotein cholesterol) or HDL-C, Type 2 diabetes, genetic risk factors and increased inflammation [19]. Therefore, on a healthcare and economic basis there is a need for innovative targets beyond LDL lowering, that allow for dose adjustment of statins as well as combination approaches with statin therapies [20]. In this regard, several approaches have been tested in clinical trials, such as combining statins with drugs that increase HDL-C [21–23]. These trials, however, failed to demonstrate beneficial effects beyond what could be achieved with statins alone. Another approach investigated treatment with the intestinal cholesterol absorption inhibitor (ezetimibe). Here, the combination of ezetimibe with simvastatin was superior to simvastatin monotherapy in lowering the risk of cardiovascular events in patients with acute coronary syndrome [24]. Other approaches include agents that lower apolipoprotein CIII (APOC3) and lipoprotein (a) (Lp(a)) [25,26]. APOC3 inhibits lipoprotein lipase–mediated hydrolysis of triglyceride-rich lipoproteins, and elevated levels of APOC3 results in the accumulation of atherogenic VLDL [27]. Lp(a) is a proatherogenic lipoprotein present in atherosclerotic but not in normal vessel walls, and Lp(a) levels are an independent risk factor for ASCVD [28]. Several strategies have been shown to reduce Lp(a) levels, including PCSK9 inhibitors [28], and antisense oligonucleotide agents [29]. Another promising target in patients with ASCVD is angiopoietin-related protein 3 (ANGPTL3), an endogenous inhibitor of lipoprotein lipase (LPL). Previously, large-scale genomics studies identified that individuals with loss-of-function variants in *Angptl3* had significantly lower LDL-C levels and reduced risk of ASCVD. Subsequent studies investigating the inhibition of ANGPLT3 using the monoclonal antibody evinacumab was associated with a significant reduction in atherosclerotic lesion size [30]. Similarly, studies investigating antisense oligonucleotide approaches targeting the *Angptl3* gene in human participants has shown promise to significantly reduce triglycerides, LDL-C and APOC3 levels [25,31].

HDL-raising strategies have also been investigated as novel therapies in ASCVD. It was observed that humans with a genetic cholesteryl ester transfer protein (CETP) deficiency have increased levels of HDL-C combined with lower levels of LDL-C [32]. These observations instigated the development of CETP inhibitors. However, despite several clinical trials investigating CETP inhibitors, the data suggest that while these approaches successfully raised HDL-C, they failed to deliver an anti-atherogenic effect, and serious adverse events were reported in some studies [33]. Similarly, pharmacological elevation of HDL-C with niacin (nicotinic acid, vitamin B3) in combination with a statin failed to further reduce CVD risk in clinical trials, despite improvements in LDL-C, HDL-C and triglyceride levels [23]. In the case of niacin, further analysis of the HDL proteome of subjects in two niacin clinical trials suggests that niacin therapy increased the levels of HDL proteins linked to increased atherosclerotic risk [34].

More recently, clinical studies have now confirmed that inflammation significantly contributes to enhanced residual risk. Therefore, reducing the extent of local inflammation at atherosclerotic plaques is seen as an attractive strategy. Therapeutic strategies directly targeting proinflammatory cytokines [e.g. tumor necrosis factor-alpha (TNF-α), interleukin-1-beta (IL-1β) neutralization, methotrexate and colchicine] have shown great promise in experimental and clinical studies in atherosclerosis [35–41]. In the recent CANTOS (Canakinumab Anti-Inflammatory Thrombosis Outcome Study) trial, treatment with an anti-IL-1β antibody (canakinumab) improved cardiovascular outcomes, independent of changes in systemic lipid levels [37]. Subsequently, the Colchicine Cardiovascular Outcomes Trial (COLCOT) and Low-Dose Colchicine 2 trial (LoDoCo2) showed that treatment with colchicine, an anti-inflammatory medication that targets the NLRP3 inflammasome/IL-1β-IL-6 axis, also leads to fewer cardiac events in patients with known ASCVD [42,43].

Non-coding RNAs (ncRNAs) have emerged as a novel class of regulatory RNAs with critical roles in the pathogenesis of atherosclerosis and vascular injury, including EC dysfunction, leukocyte recruitment and vSMC activation. A diverse array of ncRNA genes are described in the human genome, including micro-RNA (miRNA), long non-coding RNA (lncRNA) and circular RNAs (circRNA). Presently, the vast majority of these ncRNA remain poorly characterized, and we are now beginning the appreciate the interactions between different ncRNA species and the importance of mRNA–miRNA–lncRNA interaction networks in health and disease [44,45]. Here we review out current understanding of the role of ncRNA in atherosclerosis, with a primary focus on miRNA, highlighting the importance of functional investigation of key miRNA networks implicated in atherosclerosis which may ultimately lead to novel therapeutic targets.

## Micro-RNA biogenesis and bioactions

**Figure 2:**
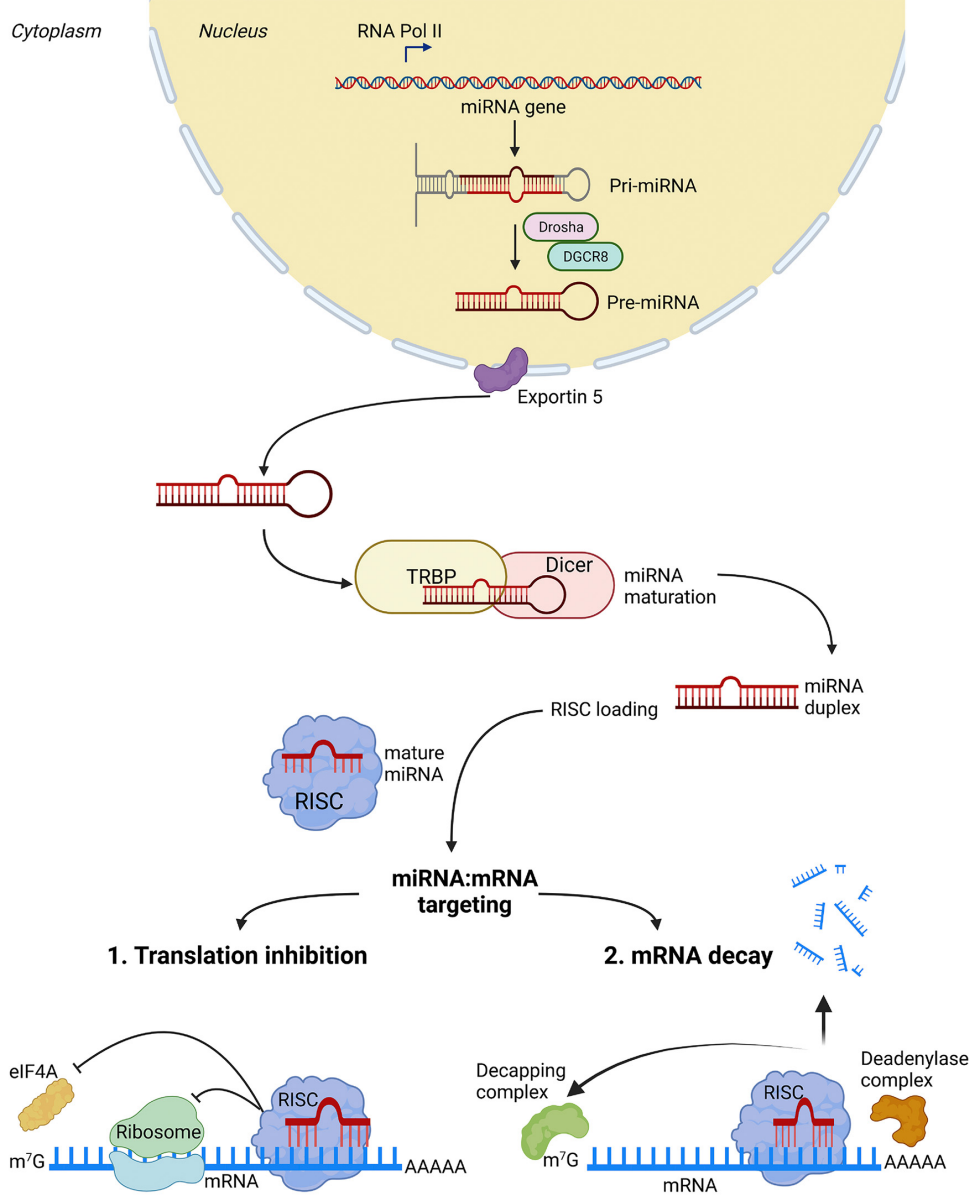
Biogenesis and canonical bioactions of miRNA miRNA genes are first transcribed via RNA polymerases II/III to generate an immature primary miRNA (pri-miRNA) transcript. Pri-miRs undergo nuclear processing by the microprocessor complex (Drosha and DGCR8 - DiGeorge syndrome critical region 8) to produce a precursor miRNA (pre-miRNA). The pre-miRNA is exported out of the nucleus into the cytoplasm by the transport protein exportin-5, and further processed by the endoribonuclease Dicer and the cofactor TAR RNA-binding protein (TRBP) into double-stranded mature miRNA duplexes. Strand selection follows, whereby the guide strand of the miRNA duplex is loaded into the RISC (RNA-induced silencing complex). The RISC is comprised of multiple protein interactants, including Argonaute proteins (AGO1–4) and the mature single stranded miRNA, which act together to target specific mRNAs. miRNA typically bind to mRNA targets in the 3′UTRs. Several mechanisms are described through which the miRNA–RISC complex can inhibit translation or initiate mRNA decay. Translation inhibition can occur through interference with ribosomal complex formation, ribosome elongation or blockade of Eif4E (eukaryotic translation initiation factor 4E) 5′ terminal cap (m7G) recognition. miRNA-mediated mRNA destabilization and decay is believed to occur through several mechanisms, including RISC-mediated deadenylation of the poly(A) tail via recruitment of a deadenylase complex, and removal of the 5′ terminal cap (m7G) via recruitment of a decapping complex. Created with BioRender.com.

Within the human genome, miRNA are found to be encoded within intergenic regions, introns of ‘host genes’ and also enriched within unique miRNA clusters [46]. Considering the number of miRNA dispersed across the human genome, and the diversity of locations, it is a significant challenge to understand the precise regulatory mechanisms that activate or repress these small RNA regulators. While our understanding of the upstream mechanisms guiding miRNA expression in cells and tissues is somewhat limited, the steps involved in miRNA biogenesis are well described [47] (Figure 2). Following biogenesis, their major role is to act as post-transcriptional negative regulators of gene expression either via cleavage and degradation of target mRNA or by inhibition of the translation process. The mode of action used to silence target genes is determined by the degree of complementarity that occurs between the miRNA complex and its target mRNA [48]. miRNA biogenesis is initiated via RNA polymerases II/III which transcribe a hairpin looped primary miRNA (pri-miRNA) molecule. Following this, the pri-miRNA undergoes nuclear processing via a multi-component microprocessor complex (Drosha/DGCR8) to produce a precursor miRNA (pre-miRNA) which retains the hairpin secondary structure. The pre-miRNA molecule is subsequently shuttled from the nucleus to the cytoplasm via the transport factor exportin-5. Within the cytoplasm, the pre-miRNA is released from exportin-5 following GTP hydrolysis and is further processed to generate a mature miRNA. This maturation step is controlled by Dicer, an endoribonuclease that recognizes the pre-miRNA and works with the cofactor (TAR) RNA-binding protein (TRBP) to produce a mature miRNA. This maturation phase involves cleavage of pre-miRNA hairpin substrates to produce short miRNA duplexes, consisting of a guide and passenger strand. miRNA duplexes bind to an Argonaute (AGO) protein complex (RNA-induced silencing complex, RISC) in a specified orientation. The bound guide strand takes the RISC to the target mRNAs, whereas the passenger strand is degraded in a process termed strand selection. Typically, the RISC complex (guide RNA and AGO protein complex) recognize sequence regions in the 3′-untranslated regions (3′-UTR) of the target mRNA sequence in a complementary manner to induce target mRNA degradation or translation inhibition. Canonical miRNA targeting requires the pairing of nucleotides 2–7 from the 5′ miRNA end (seed region) with the target mRNA 3′ UTR sequence, with perfect complementarity promoting target mRNA degradation, and imperfect complementarity promoting translation inhibition. This concept however is now deemed an oversimplification, with many non-canonical mechanisms now described which have identified roles for miRNA sequences beyond the seed region in guiding mRNA target recognition and regulation [49].

## miRNA in atherosclerosis

Atherosclerosis is driven by the dysregulation of key resident vascular cell populations (ECs and vSMCs) and infiltrating immune cells in response to a pro-atherogenic environment. Here we review the evidence demonstrating a role for miRNA in atherosclerosis, which may allow us to exploit miRNAs as novel therapeutics or clinical biomarkers that may lead to better management of atherosclerotic vascular diseases.

### Biomarker potential of miRNA in atherosclerosis

The study of circulating biomarkers that can non-invasively detect an unstable plaque in a timely manner, thus predicting the risk of advanced atherosclerosis and atherothrombotic stroke, is a significant unmet clinical need. Currently, no reliable biomarkers exist that are clinically implemented to diagnose plaque vulnerability. Thus far, perhaps the most promising developments in this space have focused on lipids and inflammatory cytokines elevated during atherosclerosis, including oxidised-LDL (ox-LDL), high sensitivity C-reactive protein, matrix metalloproteinase-9, neutrophil gelatinase-associated lipocalin, and oxidized low-density lipoprotein receptor-1 (LOX-1) [50]. From a biomarker perspective, circulating miRNA are highly stable in human plasma, existing in a form that is resistant to plasma RNase activity [51].

Several studies have investigated miRNA expression profiles in human atherosclerotic plaques as well as circulating levels, leading to the identification of dysregulated miRNA. miRNA characterization of non-coronary artery disease patients presenting with early-stage atherosclerotic plaques demonstrated an up-regulation of miR-29, miR-100, miR-155, miR-221 and miR-363, whereas down-regulation in the levels of miR-490, miR-1273 and miR-1284 [52]. A comparative study of miRNA expression between healthy thoracic arteries and aortic, carotid and femoral atherosclerotic lesions identified increased levels of miR-21, miR-34, miR-146 and miR-210 in atherosclerotic lesions [53]. Cipollone and colleagues likewise indicated that miR-100, miR-127, miR-133 and miR-145 were significantly higher in unstable symptomatic carotid lesions versus stable carotid lesions [54].The presence of miRNA as circulating biomarkers have also been studied, particularly in the context of atherosclerosis, where changes in levels of certain miRNA in blood correlate with the stage of disease progression. This was demonstrated in a comparative study between healthy and stable-coronary artery disease (CAD) patients, where circulating Let-7c, miR-145 and miR-155 levels were decreased in stable-CAD patients [55]. Furthermore, circulating levels of various miRNA such as miR-17, miR-92, miR-126, miR-145, miR-155, miR-181, miR-222 and miR-484 in blood were significantly lower in stable-CAD patients versus healthy matched subjects [56–58]. Plasma levels of miR-132, miR-150 and miR-186 have also been shown to be predictive of unstable angina [59], whereas up-regulation of miR-217 plasma levels showed significant correlation with serum lipid parameters (LDL-C and triglycerides), predicting the progression of atherosclerosis in hyperhomocysteinemia patients [60].

### miRNA regulators of vascular endothelial cell dysfunction

ECs form the inner lining of the arterial lumen and play crucial roles in maintaining vascular cell homeostasis by maintaining laminar blood flow, conferring anti-thrombotic and anti-inflammatory properties under normal cell conditions [61]. Crucially, damage to the EC layer represents the earliest detectable changes in the development of an atherosclerotic lesion. Therefore, targeting EC dysfunction represents a highly attractive therapeutic target. At a cellular level, in response to pro-atherogenic cues, defective biosynthesis of the endothelial-derived vasodilator nitric oxide (NO) and activation of inflammatory pathways in ECs are implicated in atherogenesis[3]. Proinflammatory agonists of EC activation include, TNF-α, IL1-β, ox-LDL and advanced glycation end-products. LOX-1 is a major ox-LDL receptor on ECs, with expression also detected on macrophages and vSMCs. Following exposure to proatherogenic stimuli, LOX-1 expression is increased in human atherosclerotic lesions. Ox-LDL binds to LOX-1 and stimulates ECs to produce adhesion molecules and the recruitment of leukocytes [62]. Proatherogenic and proinflammatory signals trigger the expression of EC surface adhesion molecules, e.g. vascular cell adhesion molecule-1 (VCAM1), chemokines and prothrombotic molecules [monocyte chemoattractant protein-1 (MCP-1)], tissue factor (TF), von Willebrand factor and plasminogen activator inhibitor-1. These events attract and recruit circulating immune cells (e.g. monocytes, T-cells) to the vascular wall, which can extravasate through the EC layer into the subendothelial space. These cellular events represent early pathogenic events that promote a pro-inflammatory/pro-thrombotic environment in atherosclerosis progression.

Recent findings suggest that different miRNAs at the site of vascular injury are regulated by EC function/dysfunction (Table 1) and respond to shear stress generated by disturbed flow [63]. Analysis of miRNA dysregulated during endothelial senescence, a pathological feature of atherosclerosis, identified an up-regulation of miR-34a expression in senescent human ECs, causing cell cycle arrest. This was mediated in part via miR-34a targeting of the cell cycle regulator Sirtuin 1 (*Sirt1*) [64]. Another miRNA, miR-92a which is encoded within the miR-17-92 genomic cluster on human chromosome 13 is identified as an important regulator of EC function. Animal studies in hypercholesterolemic low-density lipoprotein receptor (Ldlr) knockout mice indicate that miR-92a inhibits angiogenesis, promotes EC dysfunction and low-grade inflammation, all leading to the formation of atherosclerotic lesions [65–67]. These studies have demonstrated increased miR-92a expression in a pro-atherogenic settings, whereas antagomiRs targeting miR-92a demonstrated a protective function [67]. At a cellular level, mechanistic *in vitro* miR-92a knock-down studies in human aortic ECs demonstrated that miR-92a targets the endothelial transcription factors Kruppel like factors 2 and 4 (*Klf2/Klf4*) via interaction with specific 3′UTR sites. Here, inhibition of miR-92a led to enhanced expression of *Klf4* and subsequent inhibition of *Mcp-1*, *Vcam-1* and E-selectin (*Sele*) [68]. Similarly, inhibition of miR-92a in human vascular ECs provided evidence that miR-92a and proinflammatory mediators IL-6 and MCP-1 were up-regulated in ECs in response to shear stress, identifying suppressor of cytokine signaling 5 (*Socs5*) as a novel regulator of miR-92a expression [69].

**Table 1:** Key miRNA implicated in cellular dysfunction in atherosclerosis

miRNA	Model	Atheroprotective or Atherogenic	Proposed Function
**Endothelial cells (ECs)**			
**miR-34a**	Human ECs	Atherogenic	Regulation of contractile function, apoptosis, EC senescence (targets Sirt 1) [64]
**miR-92a**	LDLR^−/−^ mouse model Human ECs	Atherogenic	Inhibits angiogenesis, EC dysfunction, EC inflammation (targets Klf4) [65–69]
**miR-126**	ApoE^−/−^ mouse model	Atheroprotective	Regulation of inflammation (targets Icam1, Vcam1), promotes plaque regression [61,63,70,71,72]
**miR-221/222**	Endothelial progenitor cells (EPC) from CAD patients	Atherogenic	Reduced EPC differentiation. Increased in CAD patients and reveresed with atorvastatin or olmesartan medoximil [73,74]
**miR-27b**	Human atherosclerotic plaques & serum Human hepatocytes	Atherogenic	Plaque progression and development. Targets lipid-metabolism genes (Pparg, Angptl3, Gpam) [75–78]
**Vascular smooth muscle cells (vSMCs)**			
**miR-143/145**	ApoE-/- mouse model	Atheroprotective/ Atherogenic	Maintain vSMC contractile phenotype. Regulate Klf4/Klf5, Elk1, Myocd. SMC overexpression *in vivo* leads to reduced plaque size [54–56,79–81,82,83]
**miR-21**	Human carotid plaques Human vSMCS Rodent model - myointimal hyperplasia	Atheroprotective/ Atherogenic	Promotes contractile phenotype via PTEN/AKT/ ERK regulation. Conflicting reports on miR-21 mimic/anti-miR effects on atherogenesis [53,84–86,87]
**Let-7b, -7c, -7d**	Human vSMCs, Human atherosclerotic plaques (ex vivo model), ApoE^−/−^ mouse model	Atheroprotective	Inhibits vSMC migration/proliferation Attenuates proinflammatory cytokine release form human plaques Reduces plaque size *in vivo* [88–90]
**miR-26a**	Human vSMCs, HFD-fed ApoE^−/−^ mice	Atheroprotective	Promotes a vSMC contractile phenotype. Overexpression *in vivo* reduces plaque size [61,91,92]
**miR-29**	Human vSMCs, ApoE-/- mouse model	Atherogenic	Overexpression in vSMCs inhibits collagen/elastin genes Targeted inhibition in vivo reduced plaque area and enhanced plaque stability [93]
**Macrophages**			
**miR-125a-5p**	oxLDL stimulated macrophages	Atheroprotective	Inhibits proinflammatory signals (IL6, TNF-a). Targets Orp-9 [94,95]
**miR-146**	oxLDL stimulated macrophages	Atheroprotective	Inhibits proinflammatory signals (IL6, TNF-a). Targets Tlr4 [96]
**miR-155**	oxLDL/LPS stimulated macrophages. ApoE-/- mouse model	Atherogenic	Promotes M1 macrophage phenotype. Increased proinflammatory signals (IL6, IL1B, TNF-a). Suppresses anti-inflammatory signals (BCL-6, pSTAT3) Increased plaque area in aortas following miR-155 overexpression [58,97–102,103]
**miR-33**	Human macrophages, ApoE-/- mouse model LDLR-/- mouse model	Atherogenic	miR-33 reduces cholesterol efflux via repression of mitochondrial energy metabolism pathways in macrophages. Anti-miR-33 oligonucleotides reduced aortic sinus lesion area *in vivo* [104,105,106,107] Anti-miR-33 reduced atherosclerotic plaque burden, promoted macrophage efferocytosis, enhanced plaque stability [108,109]

miRNA-mediated regulation of the ox-LDL receptor LOX-1 has also been investigated in vascular cells. For example, Ang II mediated EC apoptosis is associated with reduced expression of miR-590-5p and subsequent increased expression of LOX-1, with a miR-590-5p binding site identified in the 3′UTR of the *Lox-1* gene [110]. In the same study, administration of a miR-590-5p mimic reduced *Lox-1* expression and attenuated EC apoptosis. In keeping with this, miR-590-5p mimic has also been shown to ameliorate ox-LDL-mediated EC angiogenesis, oxidative stress and proinflammatory signals, and this effect is achieved by inhibition of *Lox-1* [110,111]. In preclinical studies, intravenous delivery of a miR-590 mimic to proatherogenic ApoE^−/−^ mice demonstrated that miR-590 significantly reduced aortic atherosclerotic plaque size and lipid content, alongside reduced plasma levels of inflammatory cytokines [112].

miR-126 (miR-126-3p and miR-126-5p) is abundantly expressed in normal ECs and is believed to play a protective role against vascular inflammation by maintaining EC integrity [70,113]. miR-126 regulates inflammation by modulating the expression of adhesion molecules intercellular adhesion molecule-1 (*Icam-1*), *Vcam-1* and *Sele*, thereby promoting atherosclerotic plaque regression [61,63,70,71]. Experiments investigating miR-126 mimic administration into atherosclerotic-prone mice demonstrated a significant reduction in macrophage content and overall plaque size [72]. Several other miRNAs have been identified that enhance EC function, including miR-10a, miR146a and miR-181b. These miRNA play atheroprotective roles by repressing proinflammatory genes including *Vcam-1* and *Sele* and regulating NF-κB (nuclear factor kappa-light-chain-enhancer of activated B cells) signaling [61,63,114,115]. Other miRs such miR-27, miR-221/222 and miR-155 also influence EC function and senescence. For example, Minami et al*.* demonstrated that the miR-221/222 cluster was highly expressed in endothelial progenitor cells (EPCs) from patients with coronary artery disease, and this enhanced miR-221/222 expression effect could be reversed following 12 months treatment with atorvastatin [73]. Of note, in hypertensive patients with left ventricular hypertrophy, treatment with the angiotensin II-type1 receptor blocker olmesartan medoxomil is effective in reducing miR-221/222 levels [74]. These studies not only support a role for the miR-221/222 cluster in promoting atherosclerosis but also highlight the fact that conventional therapies may act in part via regulating miRNA expression in patients.

The miR-27 family (miR-27a/miR-27b), are encoded on human chromosomes 19 and 9, respectively. This miRNA family are highly expressed in cardiac and lung vascular tissues and ECs [116,117]. In the setting of cardiac tissue development and health, miR-27a targets thyroid hormone receptor β1, thereby enhancing the expression of β-myosin heavy chain (β-MHC), a regulator of contractility in cardiac ventricular myocytes [118]. Studies suggest the miR-27 family have utility as potential biomarkers for atherosclerosis development and progression, as well as playing a significant role in oxidative stress and lipid metabolism, along with regulation of inflammation, insulin signaling and angiogenesis in resident vascular cells [65,117]. For example, miR-27b levels were found to be increased in both plaque tissue and serum samples of patients diagnosed with peripheral artery disease (arteriosclerosis obliterans)[75]. While the precise role for the miR-27 family in EC dysfunction in atherosclerosis in limited to a few studies [76,77], there is strong evidence in the literature that this family target key cholesterol and lipoprotein metabolism genes within the liver which may have significant downstream consequences for lipid-uptake into the vascular wall during atherogenesis. An analysis of miRNA expression in liver tissue of murine models of dyslipidemia and atherosclerosis reported a significant up-regulation of miR-27b, and subsequent mechanistic studies in human hepatocytes demonstrated that miR-27b inhibits the expression of several important lipid-metabolism genes, including *Pparg*, *Angptl3* and *Gpam* [78].

### miRNA regulators of vascular smooth muscle dysfunction

vSMCs form the medial layer of blood vessels and are non-proliferative, phenotypically stable, highly contractile, quiescent cells which under stress-free conditions are terminally undifferentiated and hence remarkably plastic [119–122]. When vSMCs undergo a phenotypic switch in conditions of physiological stress, they lose their contractile markers such as ACTA2 (a-smooth muscle actin), MYH11 (smooth muscle myosin heavy chain), calponin (CNN1) and TAGLN/SM22 (Transgelin), and begin to express inflammatory and proliferative markers such as IL-6 and CD68 [119,121–124]. Until recently, the role of the vSMC layer in atherosclerosis was perhaps underappreciated and limited by our understanding of appropriate vSMC markers. This has greatly improved with advances in vSMC-lineage tracing studies and single-cell RNA-seq analysis of human carotid plaques. For example, vSMC lineage-tracing studies in murine models of atherosclerosis provides strong evidence that cell populations derived from vSMCs make a significant contribution to plaque progression in atherosclerosis, including vSMC-derived macrophages and mesenchymal stem cells. Central to the phenotypic switch of vSMCs from contractile to synthetic phenotypes is the transcription factor KLF4 which is up-regulated during atherosclerosis [125]. More recently, single cell RNA-sequencing of mouse and human atherosclerotic plaques identified multiple vSMC-derived cell types, including stem cell, ECs and monocytes, with retinoic acid implicated as a potential master regulator of these phenotypic switches [126].

Studies have shown that the vSMC phenotypic switch is potentially regulated by different miRNAs, all contributing to atherosclerotic plaque formation (Table 1) [61,63]. For example, miR-143 and miR-145 are highly expressed in vSMCs and plays a critical role in promoting and maintaining a contractile vSMC phenotype[63,79,80]. miR-143/miR-145 are co-regulated on the same genomic cluster on chromosome 5, and their expression levels are typically reduced following vascular injury or atherosclerosis. In vSMCs, reduced miR-143/miR-145 expression levels contribute to increased expression of transcription factors KLF4 and KLF5, thereby suppressing SMC contractile genes, and promoting a switch toward a synthetic phenotype [79,81,127]. Other transcription factors, such as ELK-1 and myocardin that control vSMC phenotype are also reported targets of miR-143/miR-145, which are increased as a result of vascular injury and reduced miR-143/miR-145 levels [128]. Serum response factor (SRF) and myocardin have been shown to regulate the vSMC contractile phenotype via control of miR-143/miR-145 levels [63,79,81]. Murine studies have demonstrated that miR-143/miR-145 knockdown leads to altered vascular tone and vessel wall thickness [127,82]. However, in contrast with these findings, Lovren et al. demonstrated that vSMC-specific over expression of miR-143/145 *in vivo* in a murine model of atherosclerosis (ApoE^−/−^) led to a significant reduction in overall aortic plaque size [80]. In this study, miR-145 overexpression was associated with reduced aortic expression of KLF4 and elevated myocardin expression, highlighting a mechanism through which miR-145 could modulate the vSMC phenotype [80]. Interestingly, PDGF-mediated down-regulation of miR143/145 in vSMCs leads to the development of cell matrix adhesions (podosomes), thereby facilitating vSMC migration and plasticity [129]. Taken together, these studies warrant further investigations and highlight miR-143/145 as a potential target for maintaining a contractile vSMC phenotype.

miR-21 is encoded on chromosome 17 and is considered one of the most studied miRNA across the spectrum of human disease as both a circulating biomarker and therapeutic target [130–132]. miR-21 has also been extensively explored in the context of vascular disease, with several studies demonstrating a role for miR-21 in fibrous cap stability, thereby reducing the overall risk of plaque rupture [52,133]. miR-21 was shown to be significantly down-regulated in ruptured versus stable human plaques, and administration of a miR-21 mimic into carotid plaques using ultrasound-targeted microbubbles led to a thickening of the fibrous cap, a significant reduction in lesion rupture, and enhanced vSMC proliferation [84]. In vSMCs, studies have shown that miR-21 plays an important role in maintaining a contractile phenotype via regulation of PTEN/AKT/ERK signalling pathways [85,86]. Mechanistic studies also suggest that transforming growth factor beta (TGF-β) and bone morphogenetic proteins induce a vSMC contractile phenotype via SMAD signalling and activation of miR-21 [134,135]. However, it has also been shown that miR-21 expression is up-regulated in synthetic/dedifferentiated vSMCs, all of which questions the exact role of miR-21 in atherosclerotic lesion formation [63,133]. In keeping with this, targeted inhibition of miR-21 using an anti-miR-21 coated stent prevented neointima lesion formation in a rodent model of myointimal hyperplasia [52]. These effects of miR-21 inhibition on neointima formation after vascular injury are mediated in part via up-regulation of PTEN and BCL2 [133,87]. While it is challenging to reconcile these data on miR-21, it is plausible that miR-21 promotes atherogenesis in early lesions, whereas this miRNA may exert beneficial effects in progressive atherosclerotic lesions, promoting a stable fibrous cap. Further studies will be required to elucidate the distinct roles of miR-21 in atherogenesis, and beyond the proposed roles for miR-21 in ASCVD, there are clinical trials ongoing investigating miR-21 inhibition in Alport Syndrome (NCT03373786 – RG-012), and miR-21 overexpression in diabetic wound macrophages (NCT02581098), and these data will provide important information on the consequences of long-term administration of therapies targeting this specific miRNA.

Reduced circulating and tissue expression levels of let-7 miRNA are associated with a range of inflammatory and fibrotic disorders, CVDs and cancers [55,136–143]. At least 12 distinct loci encode members of the let-7 family in humans, with let-7 family sequence and function highly conserved in mammals [142]. Let-7 miRNA are implicated in the regulation of developmental timing, cancer progression and stem cell differentiation [140,141,144–146]. Pathways targeted by let-7 miRNA include IL-6, TNF-α and NF-κB signaling [147–150]. Within distinct vascular cell populations, basal levels of Let-7 family member tend to be high, and these levels are reduced in response to proatherogenic stimuli and vascular injury [133,143,151,88]. Yu et al demonstrated that Let-7d targets KRAS thus modulating vSMC proliferation, and Let-7g expression is inhibited by the LOX-1/ROS/ERK/AP-1 pathway when stimulated with ox-LDL [65,128,89]. In line with this, Wang and colleagues demonstrated that impaired Let-7g levels led to EC dysfunction [90]. Analysis of the ox-LDL receptor LOX-1 3′UTR sequence identifies a binding site for let-7 miRNA family members, and this relationship has been further explored in vSMCs, with delivery of a let-7g mimic shown to inhibit LOX-1 expression [152]. In the same study, delivery of exogenous miR-let-7g mimic in a hyperlipidemic ApoE^−/−^ mouse model led to reduced overall atherosclerotic plaque lesion burden, believed to be mediated in part by inhibition of LOX-1. Let-7b and Let-7d have been identified as prime regulators of aortic vascular cell and tissue inflammation, with let-7 mimics attenuating vSMC proliferation, migration and inflammatory responses [88]. Of note, the development of an *ex vivo* assay to investigate the atheroprotective effects of miRNA-therapy delivery to human carotid plaque explants demonstrated that let-7d mimic modifies the inflammatory profile of these tissues, including suppression of TNF-α, IL-1β and interferon-γ [88].

A vast array of other miRNA influence vSMC proliferation, triggering a phenotypic switch, migration, and arterial calcification. For example, reduced miR-26a levels promotes vSMC differentiation, mediated in part via activation of the TGF-β signaling pathway [61,91] *In vivo* studies investigating the impact of miR-26a overexpression in HFD-fed ApoE^−/−^ mice demonstrated that increased miR-26a activity lead to reductions in hyperlipidemia, atherosclerotic lesion development and suppression of inflammatory responses [92]. Along with miR-26a, miR-146a and miR-10 regulate vSMC proliferation by targeting KLF4 transcription factor expression [61,65,153]. miR-29 is also implicated in vSMC activation in atherosclerosis, with up-regulation of this miRNA reducing the expression of extracellular proteins collagen and elastin which are present in mature vSMCs, further promoting plaque instability and rupture. Here, the targeted inhibition of miR-29 using a locked-nucleic acid strategy in a murine model of atherosclerosis (ApoE^−/−^ mice) promoted a significant reduction in atherosclerotic lesion size and further enhanced fibrous cap thickness and plaque stability [93]. miR132, miR-208 and miR-221/222 are also reported as key regulators of vSMC proliferation [65,154–156], with miR-221/222 expression up-regulated in vascular injury, resulting in vSMC proliferation and neointimal lesion formation [154].

### miRNA regulators of vascular macrophage phenotype

Atherosclerosis is driven by a pro-inflammatory environment whereby monocytes are recruited from the circulation into the vessel wall, in areas prone to endothelial dysfunction and lipid uptake [61,63]. Monocytes that differentiate into macrophages within the vessel wall retain lipoproteins and transform into lipid enriched foam cells, a classical hallmark of atherosclerosis which further contributes to plaque build-up [61,63,157]. To regulate lipoprotein uptake, macrophages express several key scavenger receptors, including SR-A1, CD36 and LOX-1. The fate of macrophages is decided by external cues, which differentiate them into M1 or classical phenotype (pro-inflammatory) or M2 (anti-inflammatory) phenotype, with M1 macrophages governing the progression of atherosclerosis [61]. Different miRNA play regulatory roles in macrophage homeostasis and polarization, potentially contributing to plaque progression or regression (Table 1) [83, 158, 159]. The scavenger receptor CD36 is a key macrophage receptor facilitating ox-LDL uptake and foam cell formation, and studies investigating CD36 deletion in atherosclerosis-prone mice demonstrate that macrophages displayed decreased proinflammatory characteristics [160,161]. miRNA regulators of CD36 have also been identified, including miR-758-5p, which directly binds the *Cd36* 3′UTR in human macrophages, thereby decreasing lipid accumulation within macrophage-derived foam cells [162]. Ox-LDL facilitates lipid retention by macrophages, which is supressed by miR-125a-5p causing down-regulation of pro-inflammatory cytokines IL-2, IL-6, and TNF-α by targeting oxysterol binding protein-like 9 (*Orp-9*), and miR-146 targeting of toll Like Receptor-4 signaling pathways (*Tlr-4*) [63,94–96]. Therefore, strategies that promote the over-expression of miR-125a-5p and miR-146 may confer atheroprotective benefit. Interestingly, several negative feedback loops have been described whereby TLR stimulation induces specific miRNAs to prevent excessive inflammatory responses. For example, miR-147 is activated in LPS-stimulated macrophages in a TLR-4/NF-κB dependent manner in order to regulate TLR-4-mediated proinflammatory pathways [163].

miR-155 is induced during the macrophage inflammatory response and is reported to be up-regulated in human atherosclerotic plaques [53,97]. miR-155 is induced by ox-LDL and proinflammatory stimuli in monocyte/macrophages, promoting an M1 macrophage polarization and a proinflammatory environment that triggers various cytokines such as IL-6, IL-1β, TNF-α and TGF-β [63,98]. Furthermore, recent *in vivo* studies have demonstrated that mir-155 expression is up-regulated in mice fed a cholesterol rich diet, with mechanistic studies in human macrophages identifying an interaction between miR-155 and the suppression of anti-inflammatory signalling molecules BCL-6 and phosphorylated STAT-3 [99,100]. While several studies have investigated the role of miR-155 in preclinical murine models of atherosclerosis, the data are conflicting, with miR-155 inhibition strategies reducing and enhancing atherosclerotic plaque development [101–103, 164]. Further studies will be required to fully characterize the role of miR-155 in atherosclerosis.

Likewise, miR-33, a miRNA encoded within an intron of the Sterol regulatory element-binding protein 2 (*Srebf2*) gene, contributes to cholesterol efflux, macrophage polarization and efferocytosis [104–106, 165, 166]. Cholesterol efflux is an essential step in the transport and removal of cholesterol from macrophages for transport to the liver. In this context, miR-33 targets ATP binding cassette transporter A1 (*Abca1*), a cholesterol transporter critical for HDL-C biogenesis, thereby preventing cholesterol efflux from macrophages and contributes to lipid retention and plaque formation [165,106]. miR-33 prevents oxidation of fatty acids, promotes oxidative metabolism facilitating M1 macrophage phenotype and anti-miR-33 treatment promotes an M2 macrophage phenotype which suppresses inflammation and promotes plaque regression [107]. More recently, silencing of miR-33 in atheroprone Ldlr^−/−^ mice demonstrated that this approach can alleviate atherosclerotic plaque development, and enhanced macrophage apoptosis and clearance [108]. Subsequent studies have also explored the targeted delivery of anti-miR-33 pH low-insertion peptide constructs to plaque macrophages, demonstrating plaque reqression and modulation of macrophage phenotype, further supporting this miR as a potential clinically relevant target [109].

Several *in vivo* studies have also demonstrated that along with miR-33, miR-128-1, miR-144 and miR-148a also target ABCA1 thereby preventing cholesterol efflux and facilitating macrophage foam cell formation [167–169]. Two isoforms of miR-27 (miR27a/b) have also been implicated in macrophage lipoprotein metabolism. miR-27 modulates several genes involved in cholesterol homeostasis such as *acat1, abca1, ldl* and *cd36* [170]. In this regard, Xie et al. demonstrated that miR-27a/b prevents atherosclerosis by targeting lipoprotein lipase which suppressed inflammation and lipid build up in human THP-1 monocytes [171].

### miRNA therapies: evidence from clinical studies

Currently, the field of miRNA research has provided ample evidence that miRNA are implicated in disease pathogenesis. Leveraging our current understanding of miRNA to make meaningful impact on clinical practice is the next step. Thus far, given their relative ease of detection and stability, most interest has centred on the biomarker potential of miRNA [172]. However, the potential of miRNA to co-ordinately regulate many pathways as ‘master regulators’ has encouraged many pharmaceutical companies to develop miRNA drug pipelines. To date, several miRNA-based therapeutics have been developed and are currently entered into different phases of clinical trials [173]. This is exemplified by the proposed clinical utility of miR-122 inhibitors to treat chronic HCV infection [174,175]. This strategy was developed following studies demonstrating that upon HCV infection, hepatocyte-enriched miR-122 interacts with the 5′ UTR of the viral genome to enhance viral RNA accumulation [176]. Anti-miR strategies targeting miR-21 (RG-012 - NCT03373786) and miR-17 (RGLS8429 – Regulus Therapeutics) are also undergoing clinical trials for the treatment of Alport syndrome and autosomal dominant polycystic kidney disease, respectively. In the cancer field, clinical trials have been performed for MRX34 as a first in class miRNA therapy in cancer. This approach mimics miR-34a, a tumor suppressor miRNA whose expression is down-regulated in patient tumours. However, a phase 1 trial of MRX34 in patients with solid tumors was halted due to serious immune-related adverse events [177,178].

Several clinical trials investigating miRNA therapies in CVD and related diseases are ongoing. Of note, miRNA-92a controls blood vessel growth and angiogenesis, and several preclinical studies have demonstrated that inhibition of miR-92a elicits beneficial responses in experimental models of vascular injury [69,179–181]. Building on these preclinical findings, the administration of a locked nucleic acid inhibitor of miR-92a (MRG-110) to healthy adults has recently been shown to be well tolerated [182], potentially paving the way for a novel miRNA therapy for CVD. Another miRNA target, miR-132 is up-regulated in cardiomyocytes in patients with heart failure and this has been shown to promote cardiac remodeling processes through regulation of cardiomyocyte autophagy and hypertrophy [183,184]. This is mediated in part via miR-132 inhibition of the anti-hypertrophic, pro-autophagic transcription factor Forkhead box O3 (FOXO3). This has led to the development of CDR132L, a miR-132 inhibitor that attenuates heart failure in preclinical models [185,186]. Building on these data, a Phase 1b randomized, double-blind, placebo-controlled study demonstrated that CDR132L was safe and well tolerated, and clinical studies are continuing [187].

## Beyond miRNA: the role of LncRNA in atherosclerosis

LncRNAs are single-stranded ncRNA species typically over 200 nucleotides in length, representing the largest class of non-coding transcripts. Recent estimates suggest up to 100,000 lncRNA are encoded in the human genome [188,189]. Several regulatory roles for LncRNA are described, including effects on mRNA transcription and stability, and interaction with regulatory proteins [190]. LncRNA have also been identified as regulators of miRNA through direct interaction, thereby inhibiting the actions of miRNA [191]. Importantly, lncRNA are amenable to overexpression and silencing studies, allowing for full *in vitro* and *in vivo* investigations into their biological functions [192]. There is also evidence that lncRNA significantly contribute to the pathogenesis of atherosclerosis, including endothelial dysfunction, leukocyte recruitment and vascular remodeling [193,194]. In recent years several studies have investigated lncRNA in human vascular cells, atherosclerotic plaques, and experimental models of atherosclerosis, highlighting novel ncRNA networks [195]. For example, key lncRNA implicated in endothelial dysfunction in atherosclerosis include *snhg12, hotair, h19, sencr, anril* and *tug1* [196–203]. Specific lncRNA regulators of smooth muscle cell function have also been identified, including *smilr, malat1, h19, neat1* and *lincrna-p21* [204–211]. Interestingly, several of these lncRNA regulate key miRNA implicated in ASCVD. For example, the lncRNA DAPK1-IT1 is up-regulated in human macrophage derived foam cells, leading to suppression of miR-590-3p, lipid metabolism dysregulation and activated inflammatory signals [212]. This is mediated in part via up-regulation of the macrophage ox-LDL receptors SRA1 and CD36, highlighting a novel mechanism through which lncRNA can target miRNA implicated in atherosclerosis.

While our understanding of lncRNA biology is limited, the evidence suggests that this class of ncRNA are an important emerging class of regulatory RNA transcripts in atherosclerosis. Going forward, lncRNA provide significant opportunities to harness their regulatory functions and develop novel RNA-based therapies. However, some challenges remain that need to be considered when investigating lncRNA. Unlike protein coding genes and miRNA, lncRNA display poor interspecies conservation and low expression levels [213]. As a result, functional analysis of human lncRNA is challenging in animal models. Similarly, the sequencing-depth offered by current single-cell RNA-sequencing platforms when analyzing human tissues is often insufficient to robustly measure low abundance lncRNA transcripts. Therefore, deep-sequencing analysis of individual cell population may offer greater insight into lncRNA biology and key lncRNA–miRNA–mRNA networks.

## Future directions and challenges for miRNA therapies in atherosclerosis

There is abundant evidence in the literature that ncRNA have important roles as regulators of vascular health, and thus represent attractive therapeutic targets. Thus far, most studies investigating miRNA in the pathogenesis of atherosclerosis have centered on either the measurement of circulating miRNA as biomarkers, mechanistic studies in vascular cells, or interventional strategies in preclinical models. The challenge now for the field is to translate these discoveries to the clinic. As we learn more about other ncRNA species such as lncRNA, there is great interest in investigating mRNA–miRNA–lncRNA interaction networks, and how these are dysregulated during the development and progression of atherosclerosis. In this regard, technical challenges remain. For example, while single-cell RNA-seq has offered insight into the coding-genome transcriptome of human aortic plaque progression, these technologies are not yet fully amenable to investigating miRNA at single-cell resolution, and low abundance lncRNA may not be robustly captured by these approaches. The current bioinformatics tools utilized by the research community for predicting ‘true’ miRNA–mRNA interactions are limited and primarily rely on the premise that the miRNA ‘seed region’ at nucleotides 2–8 is required for functional targeting (canonical seed pairing). However, it is now clear that non-canonical seed pairing mechanisms exist in mRNA-miRNAs interactions, suggesting that non-seed miRNA sequence can also impact on mRNA targeting. Therefore, methodological advancements are required to consider both canonical and non-canonical seed pairing in mRNA–miRNAs interactions [214,215].

**Figure 3:**
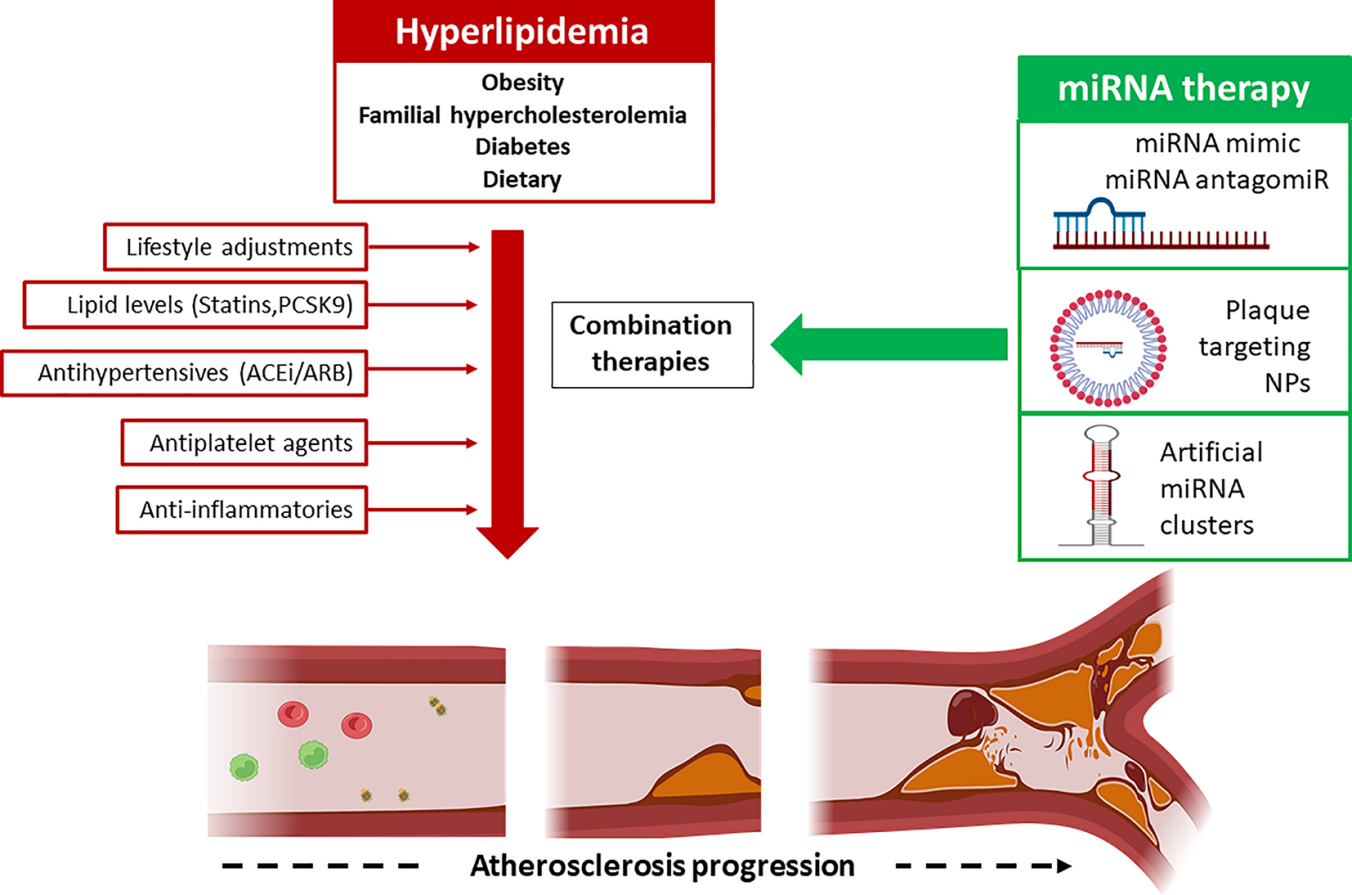
Development of novel miRNA therapies in atherosclerosis Established risk factors for hyperlipidemia and atherosclerosis include obesity, familial hypercholesterolemia, diabetes, as well as poor diet and alcohol. Current patient management strategies include LDL-C lipid-lowering agents, antihypertensives, and antiplatelet drugs alongside dietary and lifestyle interventions. In recent years, novel therapeutic approaches investigating anti-inflammatory agents have shown some therapeutic potential to reduce CVD-risk in clinical trials. Targeting of miRNA implicated in atherosclerosis pathogenesis represents another novel therapeutic avenue. Strategies currently under investigation in pre-clinical models of atherosclerosis include miRNA overexpression (miRNA mimic) and miRNA silencing (miRNA antagomir). Other areas in development include the following: (1) the targeted delivery of miRNA therapies within plaque targeting nanoparticles (NPs); (2) simultaneous targeting of multiple miRNA networks (multi-miR approach); and (3) the engineering of artificial miRNA clusters for miRNA transgene delivery of functionally associated miRNAs. Created with BioRender.com.

As outlined in this review, the evidence is clear that miRNA play an important role in the development and progression of many diseases, including ASCVD. However, several challenges need to be addressed in order to translate this class of ncRNA to clinical utility, including understanding the degradation and clearance of these agents in blood, monitoring their potential to activate the immune system, and developing efficient delivery systems that target miRNA-based drugs to the relevant site of tissue injury [216]. Targeting miRNA-based therapies to vascular lesions will likely reduce the risk of adverse events associated with systemic administration. In this regard, drug delivery strategies using nanoparticles (NPs) that target miRNA therapies to vascular lesions may overcome this problem and result in specific treatments for atherosclerosis [217] (Figure 3). In recent years, several studies have focused on the optimisation of NP delivery to vascular lesion in preclinical models, including collagen IV (Col IV) and VCAM-1 targeted nanoparticles [218–221]. While these approaches hold great promise for the future, further investigations will be required to optimize the encapsulation and delivery of miRNA-based therapies to vascular lesions to determine whether targeted-delivery confers additional atheroprotective benefit in comparison with systemic delivery of non-targeting miRNA drugs.

Simultaneous targeting of multiple miRNA networks co-regulated in disease is another approach that is being investigated (Figure 3). The concept of targeting multiple miRs is being explored in the cancer field, wherein miRNA-based agents are currently being tested for adjuvant potential and utility as chemotherapy sparing agents [222–224]. For example, dual targeting of miR-155 and miR-221 have been investigated for treatment of temozolomide-resistant gliomas, with this approach enhancing glioma cell sensitization to this agent, supporting the concept that this anti-miRNA strategy could be of therapeutic benefit [225]. Another miRNA target, miR-634 is down-regulated in glioma tissue, and restoration of miR-634 levels has been shown to sensitize glioma cells to temozolomide [226]. Similarly, simultaneous overexpression of miR-497 and miR-34a leads to enhanced suppression of the oncogene Cyclin E1 in lung adenocarcinoma cells and also in a murine tumor xenograft model [227]. Importantly, in the present study the anti-tumor effects of the dual miRNA targeting approach were greater than what was achieve by targeting individual miRNAs. This multi-miRNA-targeting strategy remains untested as an innovative approach to alleviate major pathogenic drivers of atherosclerosis. More recently, several studies have focused on the design and synthesis of artificial clusters of functionally associated microRNAs to express panels of miRNAs [228,229]. This innovative transgenic miR cluster approach allows for the study of functional synergism of multiple miRNA using a single synthetic transgene, and could potentially be exploited in preclinical models of atherosclerosis. Further investigations are warranted here. Finally, there is a need to test miRNA-based therapies alongside conventional standard-of-care therapeutics to determine whether these approaches synergise and confer additional benefit to patients (Figure 3). This approach has been explored in the cancer field where miRNA therapies have been explored as adjuvant agents alongside anti-cancer therapies. For example, co-delivery of the tumor suppressor miRNA-205 alongside gemcitabine in a pancreatic cancer ectopic tumour mouse model demonstrated significant inhibition of tumor growth, with this approach proposed as a novel strategy to overcome chemoresistance [230]. Similar studies have investigated combinations of anti-miR-21, let-7b mimic, and miR-34 mimic alongside conventional chemotherapies, with these data demonstrating improved outcomes following targeting of these specific miRNA pathways [224,231,232]. This is relevant in ASCVD as it is well established that existing medications (statins, anti-hypertensives, anti-platelet agents) can modulate systemic and tissue-specific miRNA expression [57,233–236], and indeed statin resistant is a clinically relevant problem. Going forward it will be essential to optimize miRNA profiles of established medicines for atherosclerosis alongside novel miRNA-based therapies to determine the best performing combination approaches. Based on our current understanding of optimized therapies for ASCVD, as well as ongoing developments in miRNA-based drugs, Figure 3 outlines a framework for the development of miRNA therapies in ASCVD.

In summary, miRNA represent a class of ncRNA species that have the potential to alleviate the development and progression of atherosclerosis. From a clinical perspective, the utility of miRNA as biomarkers of disease status and response to therapy is where most significant developments have been made. The challenge of off-target effects has perhaps been the greatest hurdle that miRNA-based therapies must overcome. With advances in nanomedicines and targeted delivery approaches it will be important to fully investigate miRNA mimics and inhibitors in preclinical models in order to develop novel therapies and translate these to patients.

## Abbreviations

ANGPTL3: angiopoietin-related protein 3
APOC3: apolipoprotein CIII
ASCVD: atherosclerotic cardiovascular disease
CAD: coronary artery disease
CETP: cholesteryl ester transfer protein
circRNA: circular RNA
CVD: cardiovascular disease
EC: endothelial cell
HDL-C: high-density lipoprotein cholesterol
Icam1: intercellular cell adhesion molecule-1
IL1β: interleukin 1-β
IL6: interleukin-6
LDL-C: low-density lipoprotein cholesterol
Ldlr: low-density lipoprotein receptor
LncRNA: long non-coding RNA
Lp(a): lipoprotein (a)
MCP-1: monocyte chemoattractant protein-1
miRNA: micro-RNA
ncRNA: non-coding RNA
NF-κB: nuclear factor κ-light-chain-enhancer of activated B cells
ox-LDL: oxidized-LDL
PCSK9: proprotein convertase subtilisin/kexin type 9
pre-miRNA: precursor miRNA
pri-miRNA: primary miRNA
RISC: RNA-induced silencing complex
TGF-β: transforming growth factor β
TNFα: tumor necrosis factor-α
UTR: untranslated region
Vcam1: vascular cell adhesion molecule-1
VLDL-C: very low-density lipoprotein cholesterol
vSMC: vascular smooth muscle cell
